# SPP1 facilitates cell migration and invasion by targeting COL11A1 in lung adenocarcinoma

**DOI:** 10.1186/s12935-022-02749-x

**Published:** 2022-10-20

**Authors:** Xuan Yi, Linlin Luo, Yanzhen Zhu, Hong Deng, Huitian Liao, Yang Shen, Yan Zheng

**Affiliations:** 1 Jiangxi Key Laboratory of Molecular Medicine, The Second Affiliated Hospital of Nanchang University, Nanchang, 330000 China; 2 Department of Orthopedics, The Second Affiliated Hospital of Nanchang University, Nanchang, 330000 China; 3 Second Department of Respiratory Disease, Jiangxi Provincial People’s Hospital, The First Affiliated Hospital of Nanchang Medical College, Nanchang, 330000 China; 4 Department of Anesthesia and Perioperative Medicine, The Second Affiliated Hospital of Nanchang University, Nanchang, 330000 China; 5 Pharmacy Department, Jiujiang Hospital of Traditional Chinese Medicine, Jiujiang, 332000 China; 6 Financial Department, The Second Affiliated Hospital of Nanchang University, Nanchang, 330000 China; 7 Department of Medical Genetics, The Second Affiliated Hospital of Nanchang University, Nanchang, 330000 China

**Keywords:** SPP1, LUAD, Prognosis biomarker, Invasion, Migration, COL11A1

## Abstract

**Background:**

Secreted phosphoprotein 1 (SPP1), an extracellular secreted glycol phosphoprotein, is closely related to tumor biologies, such as proliferation, migration, and invasion. However, the role and biological function of SPP1 in lung adenocarcinoma (LUAD) was still ambiguous.

**Methods:**

SPP1 expression in LUAD tissues and its associations with clinical features and prognosis was investigated using meta-analysis, immunohistochemistry (IHC) staining methods, and quantitative real-time polymerase chain reaction (qRT-PCR). Moreover, the potential mechanism related to SPP1 was identified by using the Gene Set Enrichment Analysis (GSEA) method. A series of function assays were conducted to determine the biological role of SPP1 in LUAD cell migration and invasion in vitro and vivo. The co-expressed genes of SPP1 were obtained and verified by western blot assays. The influence of SPP1 on Collagen type XI alpha 1 (COL11A1) expression and epithelial-mesenchymal transition (EMT) markers was analyzed using western blot assays.

**Results:**

The expression of SPP1 in LUAD tissues and cells was significantly higher than that in normal tissues and cells. And positively associations of SPP1 expression with TNM stage, lymph node metastasis, and invasion depth were observed. Patients with high SPP1 expression had unfavorable survival. The multivariable Cox regression analysis revealed that SPP1 expression was an independent prognostic factor of LUAD patients. Furthermore, downregulation of SPP1 could inhibit cell migration and invasion both in vitro and vivo, reduce the expression of epithelial marker (E-cadherin), and increase the expression of mesenchymal markers (N-cadherin and vimentin). Using bioinformatics and western blot assays, we confirmed that COL11A1 acted as the downstream of SPP1, and SPP1 knockdown could significantly downregulate the COL11A1 expression. Importantly, suppression of cell migration and invasion and the expression changes of EMT markers induced by SPP1 downregulation could be reversed by COL11A1 overexpression.

**Conclusions:**

SPP1 facilitates cell migration and invasion by upregulating COL11A1 expression and that acts as a potential biomarker of metastasis and prognosis for LUAD.

**Supplementary Information:**

The online version contains supplementary material available at 10.1186/s12935-022-02749-x.

## Background

Lung cancer is the most common cancer and the leading cause of cancer-related mortality all over the world, with over 2,200,000 new diagnosed cases and an approximated 1,796,144 deaths in 2020 [1]. With the morbidity persistently increased, lung adenocarcinoma (LUAD) has gradually become the commonest type of lung cancer, and its family economic burden remains considerable high compared to that of other cancers [2, 3]. Radical surgery is the only possible cure for lung cancer. However, most patients are diagnosed with advanced lung cancer or distant metastases that do not eligible for the curative surgeries. Despite the advances of targeted therapies and immunotherapies, most patients inevitably undergo chemotherapies in the first-line or subsequent treatment settings [4, 5]. Despite the early screening of lung cancer is a regular part of regular health examination, the improvement of LUAD prognosis was still inconspicuous [6]. The ultimate cause is that most patients are eventually diagnosed as suffering from terminally LUAD with local, or distant metastasis [7]. Hence, uncovering the underlying mechanisms to which gene expression changes promote migration and invasion of LUAD is important for identifying potential therapeutic targets.

SPP1, also named osteopontin, is an extracellular secreted glycol phosphoprotein. Initially, it was found to be synthesized and secreted by osteoclasts, macrophages, and epithelial cells [8]. More recently, it has been confirmed that aberrant expression of SPP1 was found in various malignancies, and it was closely related to the tumor biology, such as proliferation, migration and invasion [9–11]. Upregulated SPP1 expression can promote the metastasis of colorectal cancer, and SPP1 inhibition can improve the cisplatin chemosensitivity of cervical cancer [12, 13]. However, the role and biological function of SPP1 in LUAD was still ambiguous.

Epithelial-mesenchymal transition (EMT) is a complex and multi-factor regulation events that plays an important role in various bodily processes, such as embryonic development, organ formation, and tumor invasion and metastasis [14, 15]. Growing researches demonstrated that tumor cells undergo phenotype changes during the EMT process that allows them to acquire exercise capacity, which ultimately results in tumor invasion and metastasis [16, 17]. The changes of gene expression can affect the EMT process. Collagen type XI alpha 1 (COL11A1), a member of the collagen family, is the main component of interstitial extracellular matrix [18]. In pancreatic cancer, COL11A1 promotes the EMT through AKT pathway and associates with poor prognosis [19]. In ovarian cancer, it activates cancer-associated fibroblasts via the TGF-β3-mediated NF-κB/IGFBP2 pathway [20]. COL11A1 is participated in regulating the migration and invasion of gastric cancer cells [21]. Moreover, upregulated COL11A1 expression was found in metastatic and recurrent NSCLC, and it also acts as a target gene that involved in regulating the migration and invasion of LUAD cells [22, 23]. However, few studies were focus on the association of COL11A1 with clinical features and prognosis in LUAD.

In this study, we first illustrated that the SPP1 expression in LUAD tissues was significantly increased and was unequal with different tumor stages, invasion depth, and lymph node metastasis. SPP1 expression was an independent predictor of overall survival in LUAD. Furthermore, the downregulation of SPP1 exerted a restraining influence on the migration and invasion of LUAD cells by negatively regulating the COL11A1 expression.

## Materials and methods

### Data searching, processing and comprehensive meta-analysis

NCBI-GEO and TCGA databases were used to retrieve the LUAD datasets until May 2022. The search terms were (Lung Adenocarcinomas) OR (Lung Adenocarcinoma) OR (Adenocarcinoma, Lung) OR (Adenocarcinomas, Lung) OR (LUAD) OR (AC). The entry type and organism were restricted by series and Homo sapiens, respectively. The inclusion criteria were set as follows: datasets including tumor and normal tissues, datasets with downloadable raw or prepared data, and samples without treatment. The mean and standard deviation of SPP1 expression was calculated using SPSS 22.0 software, and the comprehensive meta-analysis of SPP1 was performed by using Stata 14.0 software. After eliminating the samples with incomplete clinical information, a total of 338 LUAD samples obtained from the TCGA-LUAD dataset were enrolled to evaluate the associations of SPP1 expression with the clinical features in LUAD. Meanwhile, the 338 samples were divided into SPP1^high^ and SPP1^low^ groups based on the median value of SPP1 expression.

### Public databases and related bioinformatic analyses

The public database Kaplan–Meier Plotter was used to evaluate the prognostic value of SPP1 and COL11A1 in LUAD (15). Moreover, we used the “survival” and “survminer” packages in R studio software to identify the potential independent prognostic factors by univariate and multivariate Cox regression analysis. The co-expressed genes (CEGs) of SPP1 in LUAD were identified (spearman's correlation ≥ 0.30, adjusted p-value < 0.05) in the cBioPortal database, and further analyzed by using the Gene Expression Profiling Interactive Analysis (GEPIA) database [24]. Meanwhile, Gene Ontology (GO) enrichment analysis was used to evaluate the biological function and potential mechanism of CEGs with the help of the Database for Annotation, Visualization and Integrated Discovery (DAVID) [25]. The results of the GO analysis were presented using R studio software. The PPI network of CEGs was constructed in the STRING database with an interaction score > 0.4 and visualized by using the Cytoscape software. Next, the three algorithms Maximal Clique Centrality (MCC), Maximum Neighborhood Component (MNC), and Degree in the cytohubba plugin were used to further identify the top 10 hub genes, and the intersection of results in the three algorithms was identified as the final hub genes. Finally, we identified the potential signaling pathways significantly associated with the SPP1 expression by performing a GSEA.

### Immunohistochemistry (IHC)

We performed a IHC to investigate the SPP1 expression in LUAD tissues and adjacent normal tissues on LUAD microarray (HLugA150CS03, Shanghai Outdo Biotech Company). Briefly, after the tissue chip was treated step by step based on the IHC protocol, all images were acquired using an inverted microscopy (XSP-C204, CIC). The expression intensity was semi-quantitatively measured by the cumulative value of the integrated optical density (IOD) and the area of the target region (Area) by using the software Image pro-plus 6.0 (Media Cybernetics, China). Furthermore, the SPP1 protein expression level was evaluated with the mean density (IOD/Area). The antibody used in IHC was anti-SPP1 (ab214050, 1:1000, Abcam).

### Cell lines and cell culture

The lung adenocarcinoma cell lines A549 (serial: TCHu150), H1299 (serial: TCHu160), and lung bronchial epithelial cells BEAS-2B (serial: GNHu27) used in this study were purchased from the Shanghai Cell Biobank of the Chinese Academy of Sciences. All cell lines were cultured in Dulbecco's modification of Eagle's medium Dulbecco (DMEM, Gibco, USA). The medium was supplemented with 10% fetal bovine serum (FBS, Gibco, USA). All cells are cultured in a cell incubator at 37 °C, with 5% CO_2_, and 95% humidity.

### Western blot

Collect the correspondingly processed lung cancer cells in an EP tube, add an appropriate amount of RIPA lysate to lyse, centrifuge, and take the supernatant. Use the Bicinchoninic Acid Protein Assay kit (BCA, Solarbio, Beijing, China) to determine the total protein concentration. After adding protein buffer, bathe in boiling water for 10 min. Take an appropriate amount of total protein in 10% sodium dodecyl sulfate–polyacrylamide gel electrophoresis (SDS-PAGE) to separate the protein, and then transfer the protein to a polyvinylidene fluoride membrane (PVDF) And sealed with 5% skimmed milk powder. Use the appropriate concentration of primary antibody to incubate overnight at 4 °C: anti-SPP1 (ab214050, 1:1000, Abcam), anti-COL11A1 (ab64883, 1:1000, Abcam), anti-E-cadherin, 1:1000 (3195S, Cell Signaling); anti-N-cadherin, 1:1000 (13116 T, Cell Signaling); anti-Vimentin, 1:1000 (5741S, Cell Signaling), GAPDH (ab8245, 1:1000, Abcam). Then, they were incubated with the appropriate concentration of secondary antibodies of the same species for 1 h at room temperature. After cleaning the membrane 3 times with 1XTBST, the protein bands were detected with an ECL luminescence reagent (Yuhengbio, Suzhou, China) and gel imaging analyzer (Bio-Rad, California, USA). Quantitative analysis of relative protein levels was performed with Quantity One software (Bio-Rad, California, USA).

### Quantitative real-time PCR (qRT-PCR)

TRIzol reagent (Invitrogen, USA) was used to extract total RNA from lung cancer cell lines, and the concentration and purity of the extracted RNA were tested. Reverse transcription was performed with Prime Script RT Reagent kit (TaKaRa, Japan). After setting up the program according to the SYBR Premix Ex Taq II kit (TaKaRa, Japan) instructions, perform real-time fluorescent quantitative PCR on a Model 7500 Real-Time PCR instrument (Applied Biosystems, CA, USA). The qRT-PCR primer sequences were listed as follows: SPP1: forward: 5ʹ-CAAATACCCAGATGCTGTGGC-3ʹ, reverse: 5ʹ-TGGTCATGGCTTTCGTTGGA-3ʹ), COL11A1: forward: 5ʹ-TGGTGATCAGAATCAGAAGTTCG-3ʹ, reverse: 5ʹ-AGGAGAGTTGAGAATTGGGATTC-3ʹ and GAPDH (forward, 5ʹ-GAAGGTGAAGGTCGGAGTC-3ʹ, reverse:5ʹ-GAAGATGGTGATGGGATTTC-3ʹ). Finally, statistical analysis is performed on the values.

### Plasmid construction and cell transfection

Empty pcDNA3.1 vector, COL11A1 pcDNA3.1 overexpression vector, small interfering RNA of SPP1 (siSPP1), negative control RNA (siCtrl), SPP1-shRNA lentiviral, and control shRNA vectors were all synthesized with the help of GenePharma Co., Ltd. (Shanghai, China). The recombinant vectors of COL11A1 were confirmed by sequencing. The siRNA and plasmid were transiently transfected into lung cancer cells using Lipofectamine 3000 (Invitrogen, USA). After 48 h incubation, the transfection effect was verified and used in subsequent experiments. The interference effect was detected by qRT-PCR and western blot.

### Cell invasion assay

The cells of each group were treated with trypsin and then resuspended inappropriate medium for counting. Working on ice, dilute Matrige gel with serum-free cold cell culture medium. Take 60 µl of the diluted gel and add it to the upper chamber of the 24-well transwell, and then incubate at 37 °C for 2 h until the gel solidifies. Add 600 µl of complete medium containing 10% FBS to the lower chamber of the transwell plate, inoculate 2 × 10^4^ lung cancer cells in the upper chamber at a density of 2 × 10^4^ cells/200 µl per well, and then place them in an incubator for 48 h. Take out the chamber, and use a cotton swab to wipe off the non-invasive cells on the upper chamber. Fix it with methanol for 20 min, then place it in 0.1% crystal violet for 20 min. Rinse with PBS to remove the crystal violet staining solution, and let it dry naturally in a ventilated place. Take pictures with an inverted microscope to observe and count the number of cells in each picture.

### Wound healing assay

The cells of each group were seeded in a six-well plate with 5 × 10^5^ cells per well. After the cells cover the entire bottom, use a 200 µl pipette tip for longitudinal scratching treatment. After washing 3 times with sterile PBS, a serum-free medium was added. Place it in a cell incubator to continue culturing, and take photos for observation at 0, 24, and 48 h.

### Cell immunofluorescence

Fix the treated cells with 4% paraformaldehyde fixative solution for 10 min at room temperature, and then remove the fixative solution. Use 0.1% TritonX-100 (polyethylene glycol octyl phenyl ether) to stand for 10 min at room temperature, then absorb the penetrant, and then wash with PBS 3 times, each time for 5 min. Add 5% BSA (bovine serum albumin) and let it stand for 1 h at room temperature. After sucking off the blocking solution, add the corresponding primary antibody, and incubate at room temperature for 2 h. After the incubation, the primary antibody was aspirated and washed with PBS 3 times for 5 min each time. After continuing to block with 5% BSA at room temperature for 1 h, add the corresponding fluorescent secondary antibody and incubate for 2 h in the dark. Aspirate the secondary antibody and wash with PBS 5 times for 5 min each time. Add DAPI (diamidino phenylindole) and incubate at room temperature for 5 min. Aspirate DAPI, washed with PBS 3 times, 5 min each time. Observe and take pictures with a fluorescence microscope.

### Statistical analysis

A comprehensive meta-analysis was utilized to evaluate the SPP1 expression between LUAD samples and normal samples and displayed on a forest plot that illustrates the standardized mean difference (SMD) and the 95% confidential interval (CI). The upper limit of 95% CI of SMD < 0 means that the SPP1 expression in LUAD tissues was lower than that in normal tissues. In contrast, the lower limit of 95% CI of SMD > 0 indicates that the SPP1 expression in LUAD tissues was higher than that in normal tissues. Cochran’s Q test and I^2^ statistics were utilized to evaluate statistical heterogeneity between studies, and a random-effect model was used if significant heterogeneity existed (I^2^ > 50% or p < 0.10). Publication bias was assessed by performing Egger’s test and Begg’s test, and the two-tailed p < 0.05 was considered to have publication bias. All the statistical analyses in this study were finished by using SPSS 22.0 software (IBM, New York, NY, USA). The student’s t-test was used to analyze the continuous variables. The chi-squared test or the Wilcoxon signed-rank test was applied to analyze the correlations of SPP1 expression with clinicopathological features. Overall survival (OS) and first progression survival (FPS) were compared using the log-rank test. Potential prognostic factors for overall survival were evaluated by performing univariate and multivariate analyses with the Cox proportional hazards regression model. The scatter plots of SPP1 mRNA and protein expression in LUAD and normal lung cell lines were performed with GraphPad Prism version 8.0. The experiment should be carried out at least three times and expressed as mean ± standard deviation (SD). p < 0.05 indicates the difference was statistically significant.

## Results

### The expression level of SPP1 was significantly increased in LUAD

**Fig. 1:**
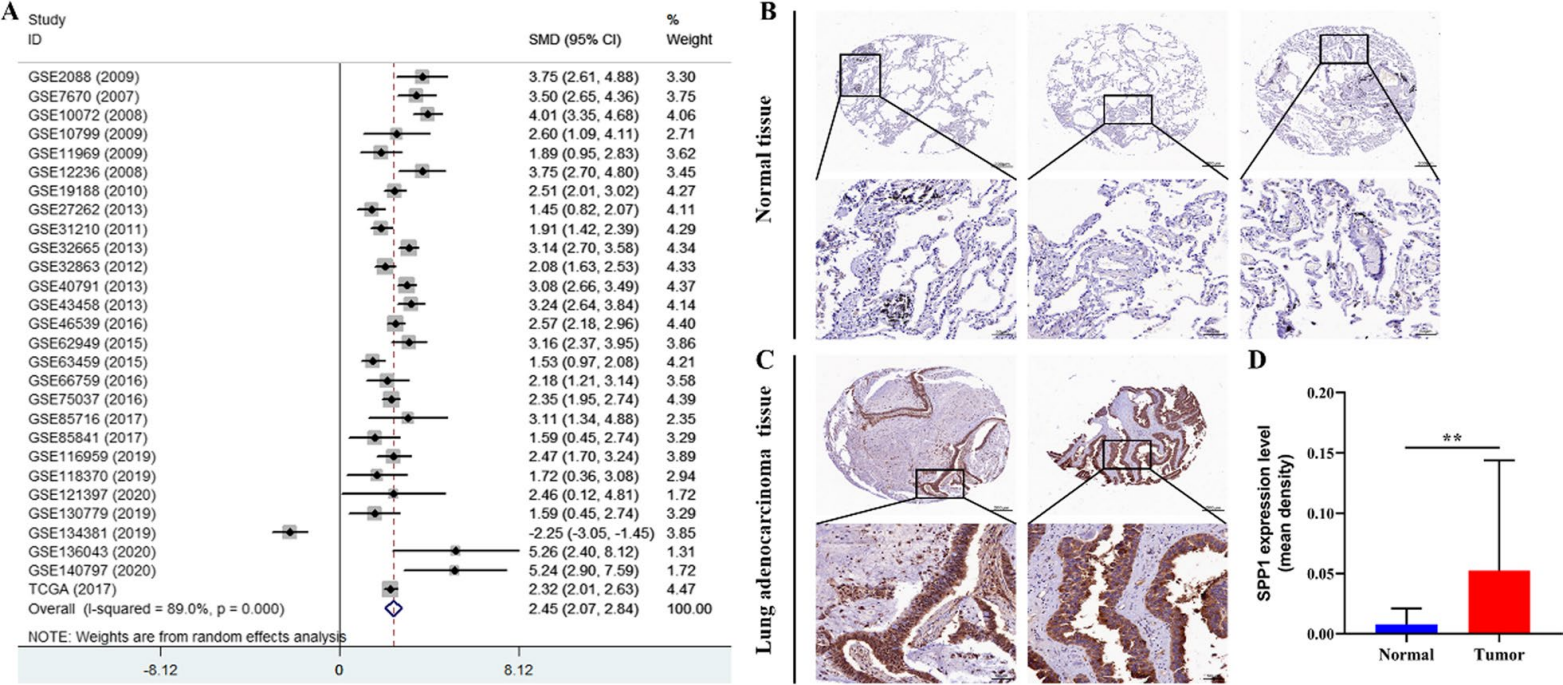
SPP1 is upregulated in LUAD. **A** Meta analysis revealed that SPP1 mRNA expression levels are higher in tumor tissue than in normal tissue; **B** representative IHC images of normal tissues; **C** representative IHC images of tumor tissues; **D** semi-quantitative analysis by IHC showed that SPP1 expression in LUAD tissues was higher than in normal tissues (*p < 0.05, **p < 0.01)

A total of 28 datasets including 1782 LUAD samples and 892 normal samples were enrolled in a comprehensive meta-analysis to evaluate the SPP1 expression and the detailed information was performed in Table 1. The meta-analysis revealed that the expression of SPP1 in LUAD samples was significantly higher than that in normal samples (SMD = 2.45, 95% CI = 2.07–2.84, I^2^ = 89.0%, p < 0.001; Fig. 1A). The results of sensitivity analysis showed that there was no specific dataset was found to have a significant connection with high heterogeneity (Additional file 1: Fig. S1A). As presented in Additional file 1: Fig. S1B, the funnel plot was generally symmetrical, and the p-value calculated by Begg’s and Egger’s tests were 0.950 and 0.921 which indicated that the publication bias of these datasets has been well controlled. Furthermore, as depicted in the results of IHC staining, only 29.55% (13/44) of normal samples were identified with high expression intensity of SPP1 (Fig. 1B), in contrast, LUAD samples with a high SPP1 intensity were shown in more than 67.35% (33/49) (Fig. 1C). The result of semi-quantitative analysis indicated that the SPP1 expression in LUAD tissues was significantly increased than in normal tissues (Fig. 1D). On the whole, these results suggested that SPP1 expression was aberrantly up-regulated in LUAD tissues.

**Table 1:** Characteristics of datasets pooled in the meta-analysis

Year	Accession	Platform	PMID	Control			LUAD		
				Number	Mean	SD	Number	Mean	SD
2009	GSE2088	GPL962	21737174	30	− 2.683	1.028	9	1.483	1.373
2007	GSE7670	GPL96	19337377	27	7.848	1.042	27	11.89	1.255
2008	GSE10072	GPL96	18297132	49	7.333	1.133	58	11.7	1.048
2009	GSE10799	GPL570	19208797	3	5.155	1.507	16	9.756	1.803
2009	GSE11969	GPL7015	21465578	5	− 1.161	0.262	90	−0.139	0.550
2008	GSE12236	GPL5188	18927117	20	4.121	1.250	20	9.185	1.444
2010	GSE19188	GPL570	20421987	65	− 2.352	1.598	45	1.695	1.626
2013	GSE27262	GPL570	25277535	25	0.772	0.076	25	1.871	1.071
2011	GSE31210	GPL570	23,028,479	20	5.705	1.484	226	8.873	1.677
2013	GSE32665	GPL6102	23591868	92	8.808	1.082	87	13.340	1.747
2012	GSE32863	GPL6884	22613842	58	7.631	0.600	58	10.140	1.596
2013	GSE40791	GPL570	23187126	100	6.791	2.183	94	12.770	1.650
2013	GSE43458	GPL6244	23659968	30	7.228	0.921	80	11.570	1.463
2016	GSE46539	GPL6883	26301798	92	− 0.745	0.697	92	0.745	0.433
2015	GSE62949	GPL8432	25650174	28	9.303	0.892	28	11.950	0.779
2015	GSE63459	GPL6883	26134223	32	8.433	1.198	33	10.550	1.544
2016	GSE66759	GPL14550	26483346	5	− 3.525	1.098	76	0.7614	2.006
2016	GSE75037	GPL6884	27354471	83	6.738	1.405	83	10.580	1.840
2017	GSE85716	GPL19612	29127420	6	5.972	1.656	6	10.580	1.289
2017	GSE85841	GPL20115	28178989	8	6.005	0.761	8	8.786	2.350
2019	GSE116959	GPL17077	31417181	11	8.277	1.938	57	12.480	1.657
2019	GSE118370	GPL570	30545439	6	7.573	1.356	6	10.840	2.325
2020	GSE121397	GPL17586	NA	3	8.534	1.403	3	10.980	0.035
2019	GSE130779	GPL20115	32869486	8	6.005	0.761	8	8.786	2.350
2019	GSE134381	GPL11532	31461552	20	8.116	2.241	20	2.704	2.567
2020	GSE136043	GPL13497	32194819	5	7.492	0.253	5	13.770	1.668
2020	GSE140797	GPL13497	32792503	7	7.463	0.893	7	12.870	1.153
2017	TCGA	HT-Seq	25079552	54	8.759	1.891	515	13.68	2.143

### SPP1 is correlated with clinical features and prognosis of LUAD

**Fig. 2:**
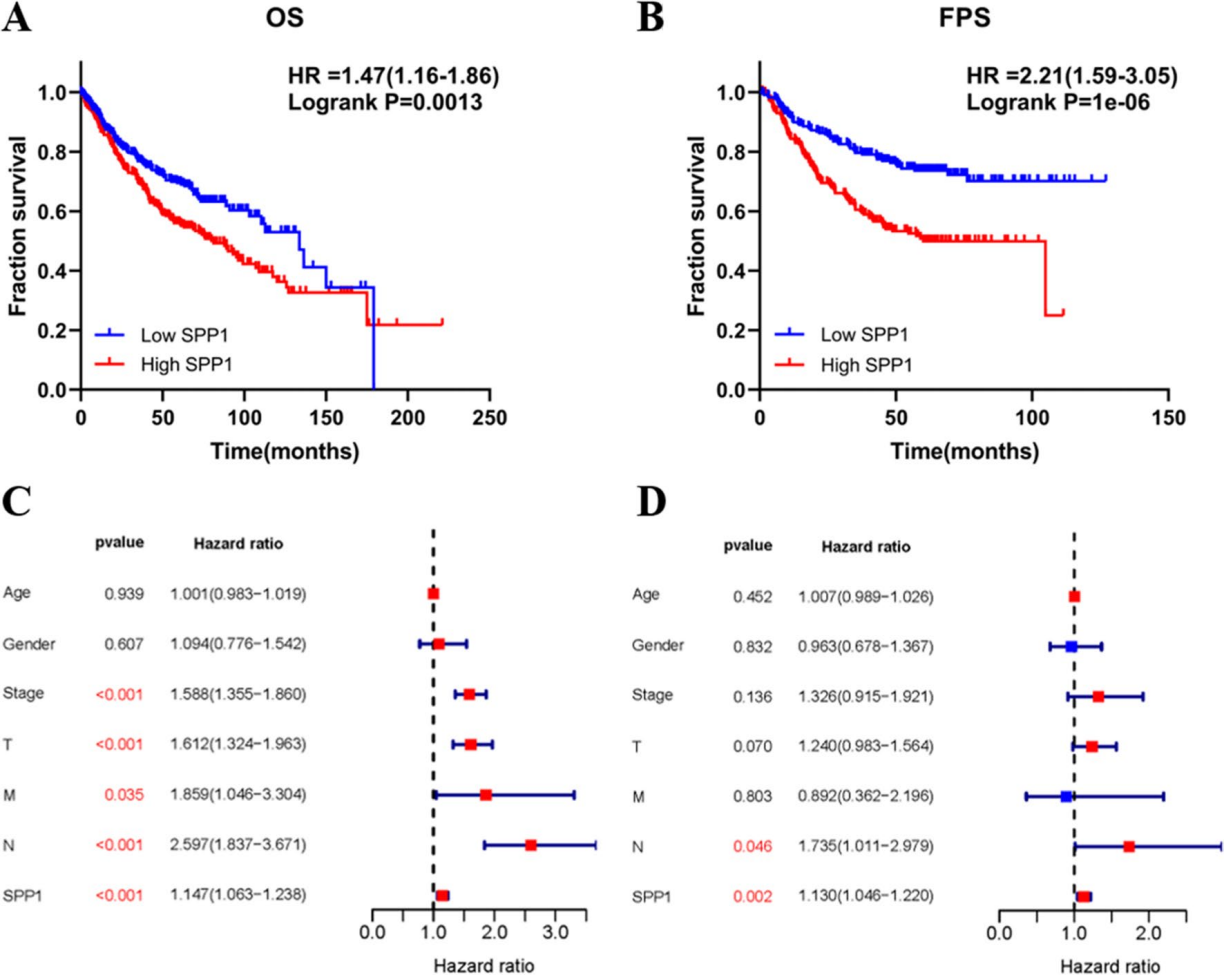
High SPP1 expression is significantly related to unfavorable prognosis in LUAD patients. **A** Overall survival time of high SPP1 and low SPP1 expression LUAD patients analyzed in the Kaplan–Meier plotter database. **B** First-progression survival of high SPP1 and low SPP1 expression LUAD patients analyzed in the Kaplan–Meier plotter database. **C** Forest plots show the HRs of different factors calculated from univariate Cox regression analysis. **D** Forest plots show the HRs of different factors calculated from multivariable Cox regression analysis

We evaluated the relationships of SPP1 with the clinical features of LUAD patients obtained from the TCGA-LUAD dataset. The proportion of patients with high SPP1 mRNA expression was unequal in different TNM stage groups (p = 0.029), lymph node metastasis groups (p = 0.006), and invasion depth groups (p = 0.024). Furthermore, as shown in Table 2, the proportion of patients with high SPP1 mRNA expression in stage II (48/79), stage III (35/60), and stage IV (10/22) was higher than that in stage I (76/177). The similar results were found in invasion depth groups (T2: 99/191, T3:21/29 vs. T1: 42/101) and lymph node metastasis groups (N1: 42/67, N2:32/53 vs N0: 95/218). As indicated in Fig. 2A, B, LUAD patients with higher SPP1 expression had a significantly poor OS (p = 0.0013) and FPS (p = 1e-06) than low SPP1 patients. Moreover, results of univariate Cox regression analysis identified that TNM stage (p = 1.00e-08), invasion depth (p = 2.01e-06), distant metastasis (p = 0.035), lymph node metastasis (p = 6.47e-08) and SPP1 expression (p = 4.13e-04) were significant prognostic factors that influence on the OS of LUAD patients (Fig. 2C). Meanwhile, in multivariable Cox regression analysis, SPP1 was further identified as an independent prognostic factor of LUAD patients (Fig. 2D). On the whole, these evidences revealed that SPP1 was a promising disadvantageous and independent prognostic indicator in LUAD.

**Table 2:** Association of SPP1 expression with clinical features in lung adenocarcinoma patients

Classification	Total	SPP1 expression		χ^2^	p-value^a^
		high	low		
Age					
≥ 60	240	118	122	0.229	0.632
< 60	98	51	47		
Sex					
Female	169	84	85	0.012	0.913
Male	169	85	84		
TNM stage					
I	177	76	101	9.038	0.029*
II	79	48	31		
III	60	35	25		
IV	22	10	12		
Invasion depth					
T1	101	42	59	9.475	0.024*
T2	191	99	92		
T3	29	21	8		
T4	17	7	10		
Lymph node metastasis					
N0	218	95	123	10.193	0.006**
N1	67	42	25		
N2	53	32	21		
Distant metastasis					
M0	316	159	157	0.194	0.659
M1	22	10	12		

^a^Chi-square test

*p < 0.05, **p < 0.01

### The enriched signaling pathways was identified by GSEA

**Fig. 3:**
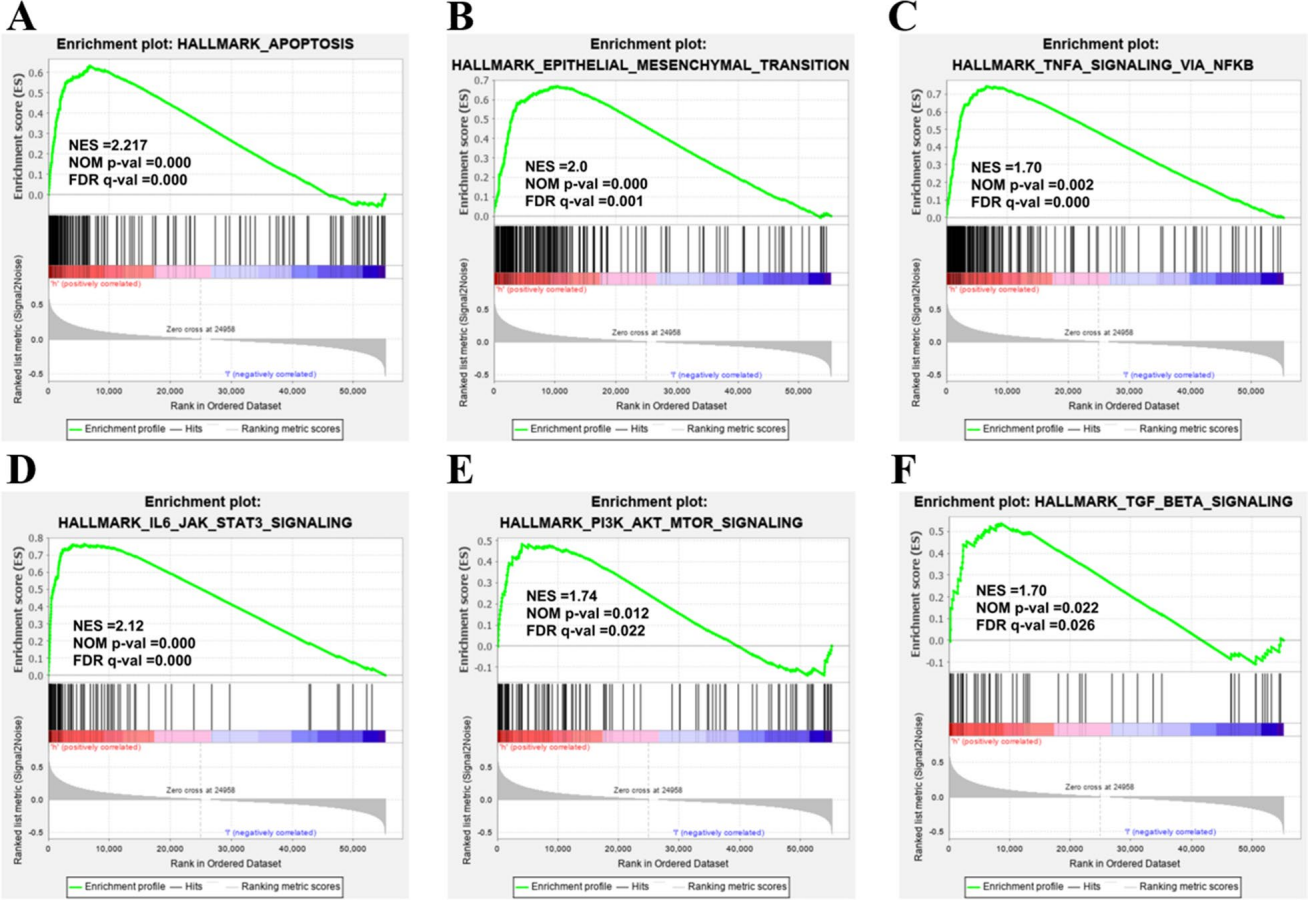
Signaling pathways enriched in high SPP1 expression phenotype. **A** Apoptosis; **B** EMT; **C** TNFA signaling via NF-KB; **D** IL6-JAK-STAT3 signaling; **E** PI3K-AKT-MTOR signaling; **F** TGF-BETA signaling; NES, normalized enrichment score

To further investigate the potential mechanisms obligated to SPP1-mediated LUAD progression, the GSEA was performed. The patients in TCGA-LUAD dataset were divided into high-SPP1 and low-SPP1 groups based on the median value of SPP1 expression. According to the criteria we set, a total of 32 signaling pathways were significantly enriched in the SPP1 high phenotype (Table 3). Moreover, we found that these enriched pathways were mainly associated with oncogenesis and tumor metastasis, such as HALLMARK_EPITHELIAL_MESENCHYMAL_TRANSITION, HALLMARK_TNFA_SIGNALING_VIA_NFKB, and HALLMARK_APOPTOSIS (Fig. 3).

**Table 3:** Signaling pathways enriched in high expression of SPP1

Gene set names	Size	NES	NOM p-val	FDR q-val
HALLMARK_COAGULATION	138	2.30	0.000	0.000
HALLMARK_IL2_STAT5_SIGNALING	199	2.29	0.000	0.000
HALLMARK_COMPLEMENT	200	2.26	0.000	0.000
HALLMARK_INFLAMMATORY_RESPONSE	200	2.22	0.000	0.000
HALLMARK_APOPTOSIS	160	2.22	0.000	0.000
HALLMARK_ALLOGRAFT_REJECTION	196	2.19	0.000	0.000
HALLMARK_INTERFERON_GAMMA_RESPONSE	198	2.15	0.000	0.000
HALLMARK_TNFA_SIGNALING_VIA_NFKB	199	2.12	0.002	0.000
HALLMARK_IL6_JAK_STAT3_SIGNALING	87	2.12	0.000	0.000
HALLMARK_KRAS_SIGNALING_UP	199	2.06	0.000	0.000
HALLMARK_P53_PATHWAY	196	2.03	0.002	0.001
HALLMARK_INTERFERON_ALPHA_RESPONSE	96	2.03	0.000	0.001
HALLMARK_HYPOXIA	197	2.01	0.000	0.001
HALLMARK_EPITHELIAL_MESENCHYMAL_TRANSITION	198	2.00	0.000	0.001
HALLMARK_XENOBIOTIC_METABOLISM	198	1.87	0.000	0.008
HALLMARK_APICAL_JUNCTION	200	1.86	0.004	0.009
HALLMARK_BILE_ACID_METABOLISM	112	1.82	0.000	0.012
HALLMARK_PEROXISOME	104	1.82	0.002	0.012
HALLMARK_ANGIOGENESIS	36	1.81	0.004	0.012
HALLMARK_REACTIVE_OXYGEN_SPECIES_PATHWAY	49	1.75	0.012	0.021
HALLMARK_PI3K_AKT_MTOR_SIGNALING	105	1.74	0.012	0.022
HALLMARK_GLYCOLYSIS	199	1.72	0.012	0.024
HALLMARK_TGF_BETA_SIGNALING	54	1.70	0.022	0.026
HALLMARK_MTORC1_SIGNALING	197	1.69	0.020	0.027
HALLMARK_HEME_METABOLISM	195	1.68	0.002	0.029
HALLMARK_APICAL_SURFACE	44	1.66	0.008	0.031
HALLMARK_ANDROGEN_RESPONSE	99	1.64	0.038	0.036
HALLMARK_ESTROGEN_RESPONSE_LATE	198	1.64	0.012	0.035
HALLMARK_MYOGENESIS	199	1.63	0.018	0.036
HALLMARK_UV_RESPONSE_UP	156	1.62	0.010	0.036
HALLMARK_ADIPOGENESIS	198	1.62	0.018	0.035
HALLMARK_CHOLESTEROL_HOMEOSTASIS	73	1.62	0.037	0.035

Gene sets with NOM p-value < 0.05 and FDR q-value < 0.2 are considered as significant

*NES* normalized enrichment score, *NOM p-val* nominal p-value, *FDR q-val* false discovery rate q-value

### Downregulation of SPP1 inhibits LUAD cells migration, invasion and EMT

**Fig. 4:**
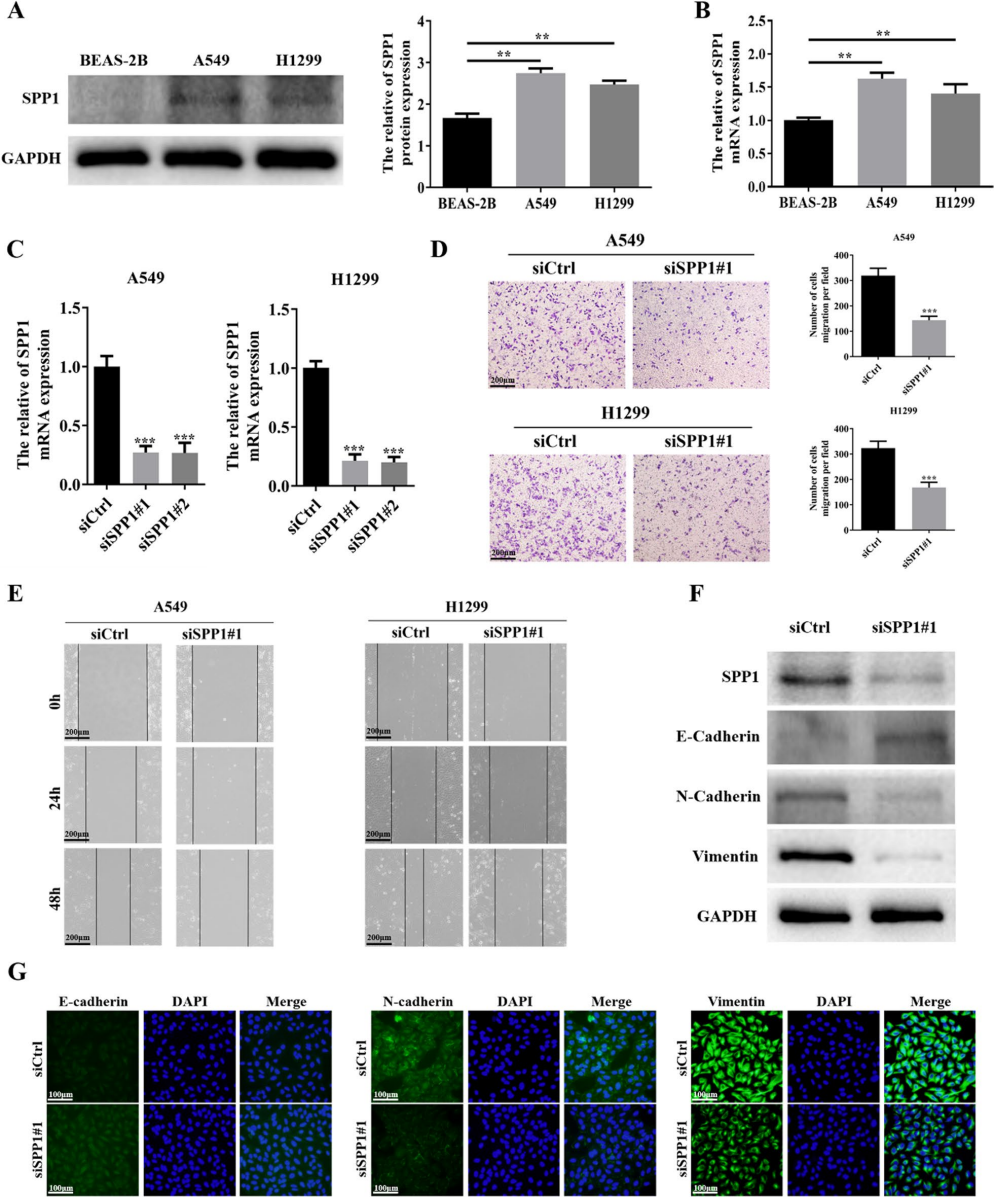
SPP1 promotes cells migration, invasion and EMT of LUAD in vitro. **A**, **B** The expression levels of SPP1 in LUAD cells A549 and H1299 and normal lung epithelial cells BEAS-2B were analyzed by Western blot and QRT-PCR(**p < 0.01); **C** the qRT-PCR experiment verified the interference effect of siSPP1 (***p < 0.001); **D**,** E** cell invasion and wound healing assays verify the effect of down-regulation of SPP1 on the migration and invasion ability of LUAD cells (***p < 0.001, **p < 0.01); **F** western blot analysis of changes in the expression levels of EMT-related proteins in A549 cells after down-regulation of SPP1; **G** the expression of EMT-related proteins in A549 cells after SPP1 downregulation was analyzed by immunofluorescence assay

The qRT-PCR and western blot were conducted to determine the expression levels of SPP1 mRNA and protein in cell lines. The results indicated, in agreement with previous results, that both mRNA and protein levels of SPP1 were remarkably increased in A549 and H1299 cells compared to that in BEAS-2B cells (Fig. 4A, B). To further investigate the potential biological function of SPP1 in LUAD, functional experiments were conducted in LUAD cell lines. A549 and H1299 with high endogenous SPP1 expression were selected to construct the cell model of SPP1 deficiency by transfecting SPP1 target-specific siRNA. After 48 h transfection, the qRT-PCR and western blot assays were used to confirm the knockdown efficiency of SPP1-siRNA (Fig. 4C, F). The SPP1 target-specific siRNA siSPP1#1 was selected for further experiments in vitro. The results of cell invasion assays showed that down-regulation of SPP1 significantly inhibited the invasion of LAUD cells (Fig. 4D). And the results of wound healing assay uncovered that decreased SPP1 expression induced the inhibition of cell migration ability (Fig. 4E). EMT has been demonstrated to be closely associated with tumor migration and invasion. Meanwhile, the results of GSEA analysis suggested that SPP1 was positively related to EMT (Fig. 3B). Therefore, to further demonstrated whether the SPP1 deficiency could affect the EMT process, cell immunofluorescence and western blot assays were conducted. The western blot analysis uncovered that down-regulation of SPP1 could significantly decrease the E-cadherin expression, and increase the expression of N-cadherin and vimentin (Fig. 4F). Moreover, similar results were carried out in immunofluorescence staining of the related marker of EMT (Fig. 4G). Altogether, these experimental results turned out that SPP1 suppression inhibited the migration and invasion of LUAD cells by affecting the EMT process.

### *Downregulation of SPP1 inhibits LUAD pulmonary metastasis *in vivo

**Fig. 5:**
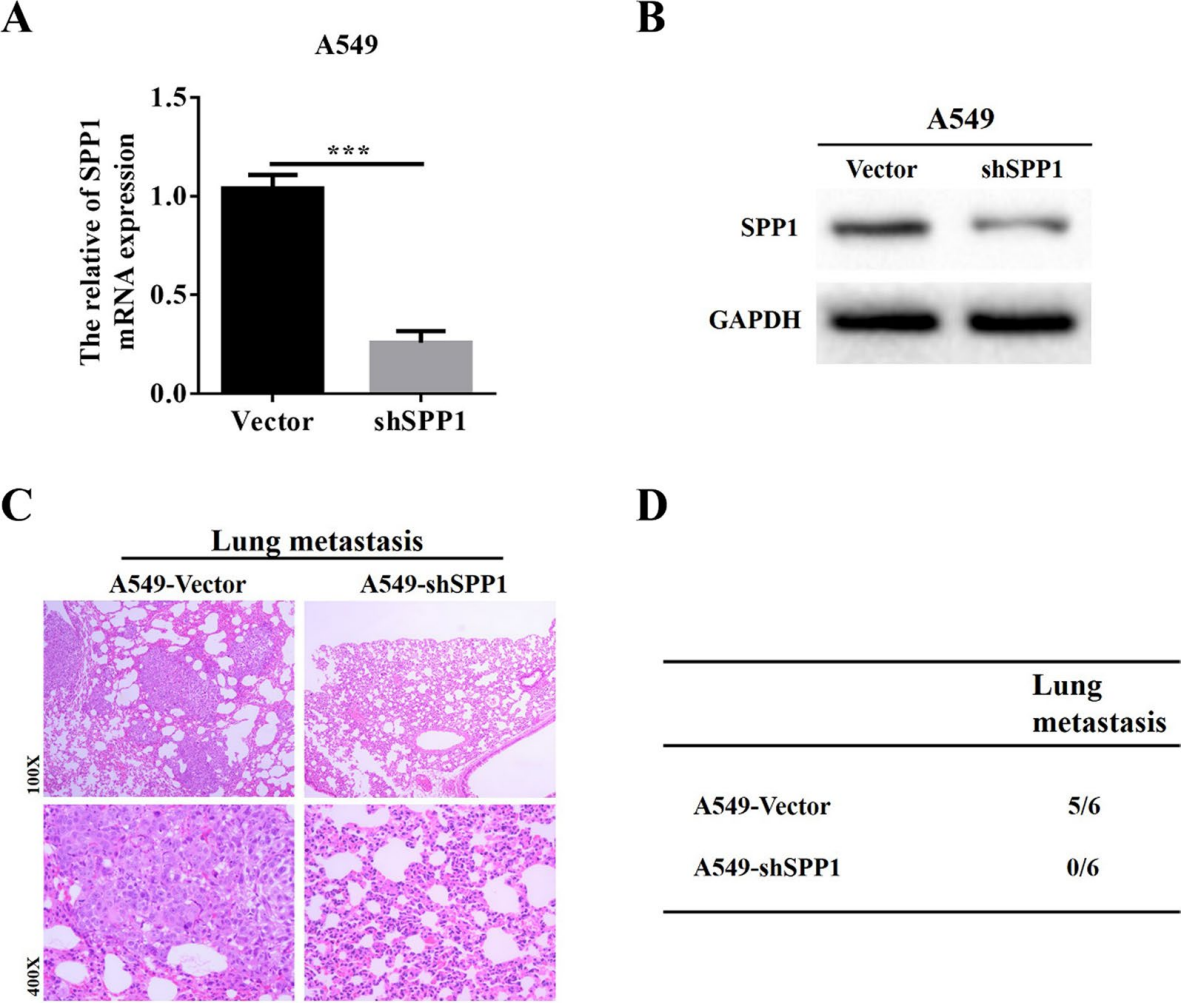
Downregulation of SPP1 inhibits LUAD pulmonary metastasis in vivo. **A**, **B** qRT-PCR and Western blot to verify the downregulation efficiency of SPP1 in stable A549 cells (***p < 0.001); **C** pathological analysis of HE staining of lung tissue of nude mice in A549-Vector and A549-shSPP1 groups; **D** incidence of lung metastases in nude mice in A549-Vector and A549-shSPP1 groups

Cell experiments show that inhibiting the expression of SPP1 can significantly reduce the invasion, migration and EMT ability of LUAD cells in vitro. Therefore, we further explored the effect of SPP1 down-regulation on the lung metastatic ability of LUAD by lung metastasis experiments in vivo. We transfected A549 cells with lentivirus-SPP1-shRNA and lentivirus-SPP1-Vector to construct stable cell lines. The effectiveness of SPP1 downregulation was verified by qRT-PCR and Western blot (Fig. 5A, B). The above cells (1X10^6^) were injected into the tail vein of nude mice (male BALB/c-nu/nu, 6–8 weeks old) to establish a lung metastasis model. One month later, the lung tissues of the two groups of mice were stained with HE. Histological analysis showed that there were no obvious lung metastases in mice in the shSPP1 group, while 5 cases in the control LUAD cell group developed obvious lung metastases (Fig. 5C, D). These results further confirmed that inhibition of SPP1 expression could inhibit LUAD invasion and metastasis in vivo.

### Screening of the CEGs and identification of the hub genes

**Fig. 6:**
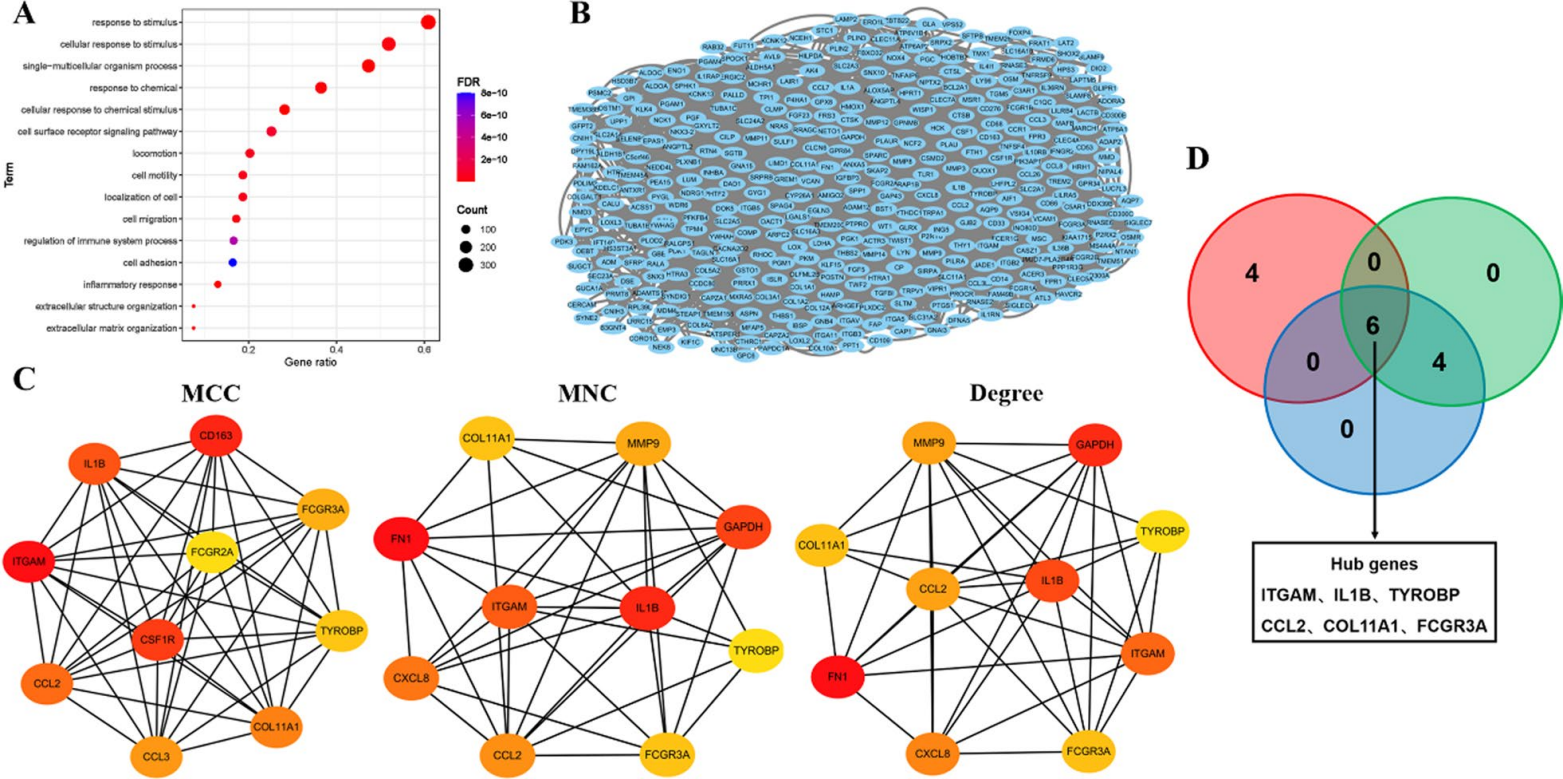
Screening of the CEGs and identification of the hub genes. **a** GO analysis of the CEGs; **b** PPI network of CEGs, containing 495 nodes and 2523 edges. The nodes represent different proteins, and each edge indicates one protein–protein interaction; The value of the gene degree is represented by the size of the node; **c** a total of three cluster modules identified by three algorithms in Cytohubba plugin; **d** the Venn diagram represents the 6 hub genes intersected by three algorithms

To further identify genes related to SPP1 in LUAD, we used the Co-expression module in the cBioPortal database to identify CEGs. While the cutoff value (spearman’s correlation > 0.3, FDR < 0.05) was set, a total of 509 CEGs were identified. Next, GO analysis was conducted to evaluate the enriched biological processes of the CEGs. Similar to the experimental results in vitro, the GO analysis showed that the CEGs of SPP1 were mainly enriched in pathways, including cell migration, cell adhesion, extracellular matrix organization, and cell location (Fig. 6A). In the meanwhile, the 509 CEGs were imported into STRING for PPI network analysis. A PPI network containing a totally of 495 nodes and 2523 edges was acquired and visualized with Cytoscape (Fig. 6B). In addition, three algorithms in the Cytohubba plugin were used to determine the top 10 hub genes respectively, and 6 hub genes were identified by crossing all the results (Fig. 6C, D). The 6 hub genes included ITGAM, IL1B, TYROBP, CCL2, COL11A1, and FCGR3A.

### COL11A1 acts a downstream of SPP1 and promotes the cell migration, invasion and EMT

**Fig. 7:**
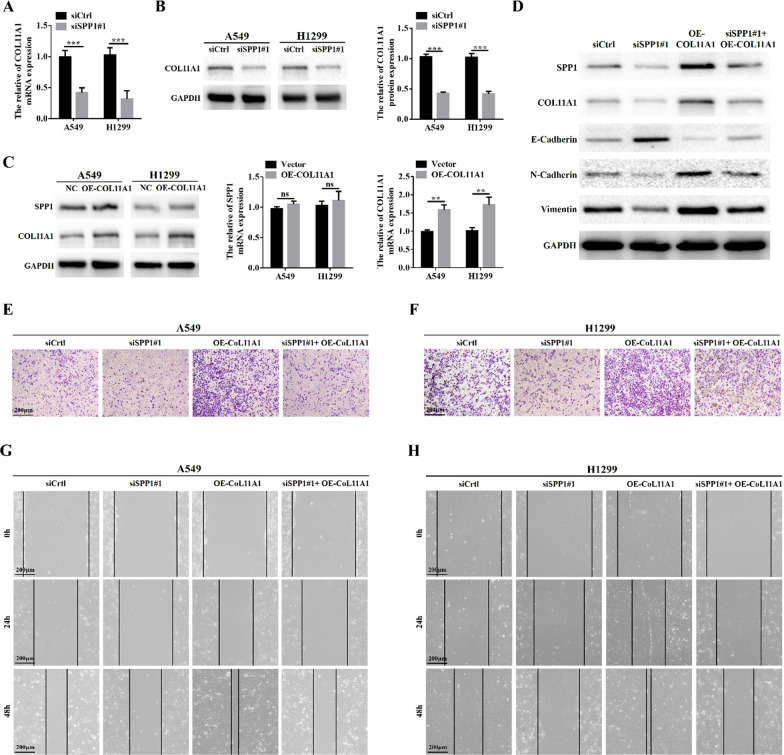
COL11A1 acts downstream of SPP1 and promotes cell migration, invasion, and EMT. **A**, **B** Changes in mRNA and protein levels of COL11A1 after down-regulation of SPP1 in LUAD cells by qRT-PCR and Western blot assays (***p < 0.001); **C** western blot and qRT-PCR assays were used to detect the changes of SPP1 protein and mRNA levels after COL11A1 overexpression in LUAD cells, respectively (**p < 0.01); **D** the expression of SPP1 was simultaneously inhibited in A549 cells that stably overexpressed COL11A1 and the expression changes of EMT-related proteins were analyzed by Western blot; **E**, **F** stable overexpression of COL11A1 while inhibiting the expression of SPP1, transwell assay was used to analyze the change of invasive ability of LUAD cells; **G**, **H** inhibition of SPP1 expression in LUAD cells stably overexpressing COL11A1 and analysis of changes in migratory ability by wound healing assay

Among the 6 hub genes, COL11A1 was confirmed to be positively associated with SPP1 in LUAD (Additional file 2: Fig. S2A). Furthermore, we further analyzed the COL11A1 expression in LUAD tissues and normal tissues. As shown in Additional file 2: Fig. S2B, C, COL11A1 expression in LUAD tissues was significantly higher than that in normal tissues, and it was also significantly correlated to the TNM stage. LUAD patients with high expression of COL11A1 had a poor OS than low COL11A1 patients (Additional file 2: Fig. S2D). Thus, COL11A1 was selected for further analysis in vitro. First, the qRT-PCR and western blot analyses showed that the expression level of COL11A1 mRNA and protein was significantly reduced in the siSPP1 group compared to that in the sictrl group (Fig. 7A, B). On the contrary, the COL11A1 overexpression did not change the expression of SPP1 (Fig. 7C). COL11A1 overexpression could reverse the suppression of the EMT process induced by SPP1 knockdown (Fig. 7D). Furthermore, Overexpression of COL11A1 also eliminated the inhibition of LUAD cell migration and invasion caused by SPP1 downregulation (Fig. 7E–H). In all, these results suggested that COL11A1 is an important functional element of SPP1 participating in the migration and invasion of LUAD cells.

## Discussion

LUAD is a common type of lung cancer and accounts for a great number of tumor-related death. The high recurrence and metastasis rate is the main cause of death in lung cancer patients that makes regular follow-up become effective measures for LUAD management, which bring a huge strain on the family economy [5, 6]. Despite breakthroughs in diagnosis methods and treatments for LUAD in the past two decades, the overall survival and prognosis of LUAD patients were still unsatisfactory. Therefore, finding a potential biomarker for predicting the LUAD prognosis or even preventing the tumor progression, invasion and metastasis is crucial to decreasing the recurrence rate and case fatality rate of LAUD.

Many studies have reported that SPP1 is participated in the invasion and metastasis of solid tumors [9–12]. Patients with high SPP1 overexpression have a poor prognosis in lung cancer [26]. However, its role in LUAD was hitherto still unknown. In this study, a comprehensive meta-analysis indicated that the SPP1 expression in LUAD tissues and cells were significantly higher than those in normal lung tissues and cells. Moreover, the high SPP1 expression was closely related to clinical features, such as TNM stage, invasion depth, and lymph node metastasis. Patients with high SPP1 expression have a worse OS and FPS compared to the low SPP1 patients, and high SPP1 expression was demonstrated to be an independent prognostic factor of OS. It indicates that SPP1 is engaged in the tumorigenesis and progression of LUAD.

SPP1 is a secreted non-collagenous protein that plays an important role in tumourigenesis and metastasis [27]. Previous studies have reported that SPP1 overexpression promotes the proliferation and restrains the apoptosis of ovarian and colon cancer cells [28, 29]. More recently, SPP1 has been reported to be involved in tumorigenesis by regulating EMT, and this may be a new mechanism of SPP1 mediated tumor invasion and metastasis. SPP1 expression was significantly up-regulated in colorectal cancer, and it promoted the cells invasion and metastasis of colorectal cancer by activating EMT process [12]. However, the molecular mechanism of SPP1 in cells invasion and metastasis of LUAD was still unclear.

Local recurrence and distant metastasis of tumor are the main causes of death in LUAD patients, and indicate failure of treatment [30]. Therefore, exploring the molecular mechanisms of invasion and metastasis in LUAD is an urgent need for clinical management. The enhancement of tumor cells migration gained from the EMT process is the main manifestation and mechanism of tumor metastasis, accompanied by changes in the expression of cell markers, such as E-cadherin (epithelial marker), N-cadherin and vimentin (mesenchymal marker) [14–16]. In this study, we found that downregulation of SPP1 suppressed the migration and invasion ability of LUAD cells in vitro, and similar inhibitory effect on migration and invasion ability was observed in vivo. Furthermore, EMT pathway was enriched in high SPP1 expression phenotype. SPP1 knockdown could significantly downregulate the E-cadherin expression, and upregulate the expression of N-cadherin and vimentin. Therefore, these results demonstrated that SPP1 promotes the LUAD cells migration and invasion through regulating the EMT.

The acquisition of motility and invasiveness by cancer cells involves the EMT and the activation of tumor microenvironment (TME), both of which can promote the invasion, metastasis and drug resistance of cancer cells [31]. Cancer-associated-fibroblasts (CAFs), the core functional components of the TME, promotes the EMT process by secreting large amounts of COL11A1 collagen [32]. COL11A1 is known to critically regulate EMT [33, 34]. Previous studies reported that COL11A1 modulates the NF-κB/IGFBP2/TGF-β3 cascade to promote EMT and activate the CAFs in ovarian cancer [20]. Similarly, Shen et al. found that COL11A1 promotes EMT, migration and invasion of NSCLC cell lines in vitro [23]. However, the upstream mechanism by which COL11A1 regulates EMT of LUAD cells has not been fully illustrated. In this study, COL11A1 was identified as hub gene of SPP1 and prognostic biomarker of LUAD patients. Furthermore, we also observed that both the mRNA expression levels of SPP1 and COL11A1 were higher in LUAD, and a positively correlation was existed in expression levels. SPP1 knockdown could reduce the mRNA and protein expression of COL11A1, whereas the COL11A1 overexpression did not change the expression of SPP1. It means that COL11A1 was acted as downstream gene of SPP1 in promoting the EMT and metastasis of LUAD. Using in rescue experiments, the association among SPP1, EMT and COL11A1 in LUAD was investigated. The results demonstrated that SPP1 knockdown could downregulated the COL11A1 expression and inhibited cells migration, invasion and EMT of LUAD. Moreover, rescue experiments indicated that the inhibition on cells migration, invasion and EMT induced by SPP1 downregulation could be reverse by COL11A1 overexpression. These results suggested that SPP1 potentially facilitates EMT through regulating the COL11A1 in LUAD cells.

## Conclusion

On the whole, SPP1 is a potential predictor of metastasis and prognosis for LUAD patients, which may contribute to identify the high-risk patients who need early, systematic, and precise treatments. additionally, SPP1 acts as a promotor and accelerator of EMT through upregulating COL11A1, thus resulting in the cells migration and invasion in LUAD.

## Supplementary Information

**Additional file 1: Fig. S1.** Analysis of Sensitivity and publication bias in meta-analysis. A Sensitivity analysis of selected datasets. B A Begg’s funnel plot with 95% confidence limits.

**Additional file 2: Fig. S2.** COL11A1 acts a tumor promotor of LUAD. A COL11A1 was positively associated with SPP1 in LUAD; B COL11A1 expression was significantly higher in LUAD tissues than normal tissues (*p <0.05, **p <0.01); C COL11A1 was significantly correlated to the TNM stage of LUAD; D patients with high COL11A1 expression had a poor OS than low COL11A1 patients.

## Data Availability

The publicly available datasets presented in this study can be found in online databases: the TCGA-GDC database (https://portal.gdc.cancer.gov/), the NCBI Gene Expression Ominibus database (https://www.ncbi.nlm.nih.gov/gds), the cBioPortal database (https://www.cbioportal.org/), the GEPIA database (http://gepia.cancer-pku.cn/).
