# Supplementary figures and images for: SPP1 facilitates cell migration and invasion by targeting COL11A1 in lung adenocarcinoma
Source: Cancer Cell Int. 2022 Oct 20;22:324. doi: 10.1186/s12935-022-02749-x (PMC9583566; doi:10.1186/s12935-022-02749-x)

Supplementary Fig. 1


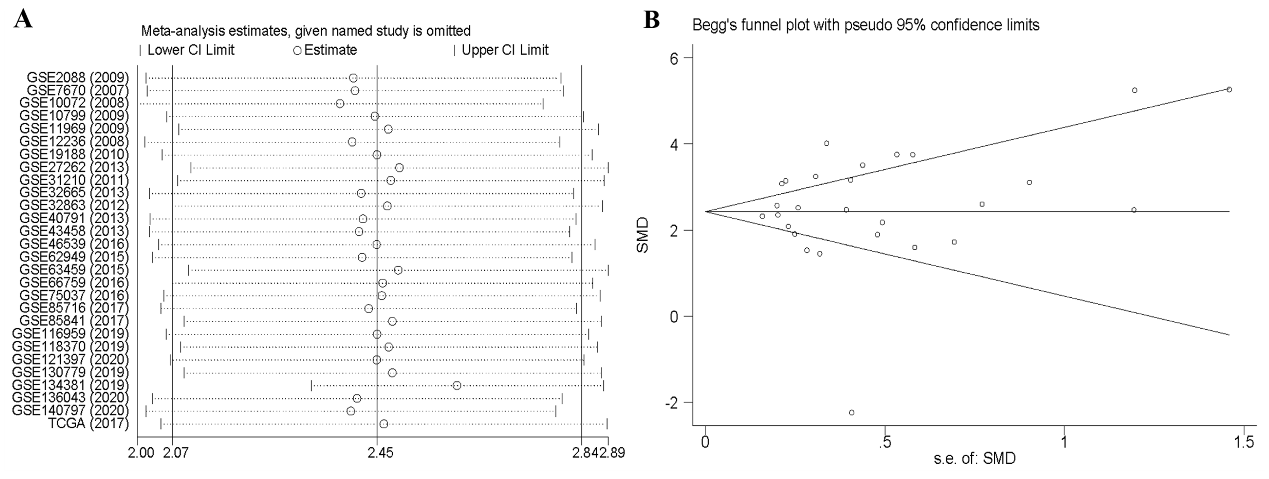

Supplement: Supplementary file 1 — Additional file 1: Fig. S1. Analysis of Sensitivity and publication bias in meta-analysis. A Sensitivity analysis of selected datasets. B A Begg’s funnel plot with 95% confidence limits. [file 12935_2022_2749_MOESM1_ESM.docx]

Supplementary Fig. 2


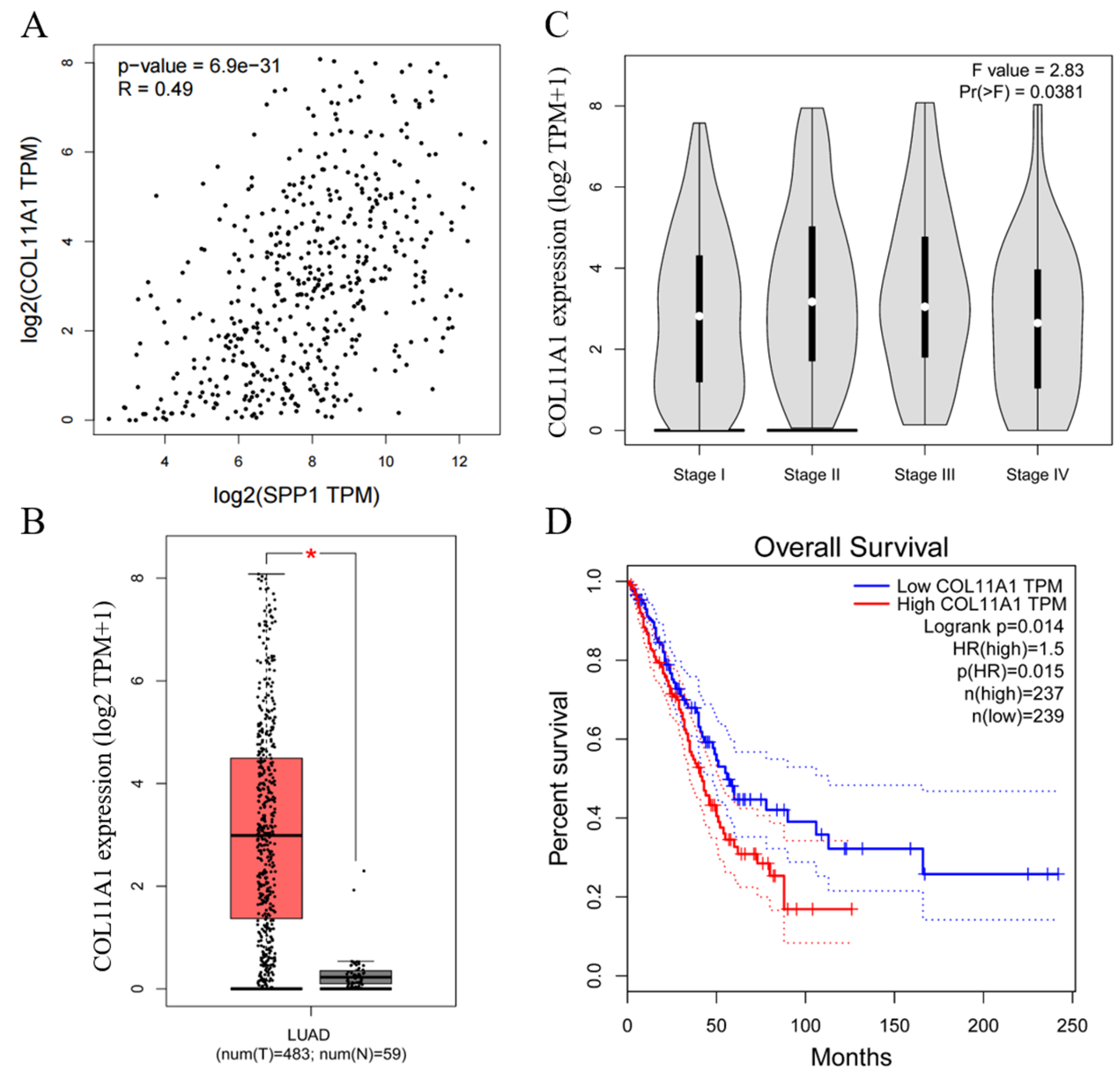

Supplement: Supplementary file 2 — Additional file 2: Fig. S2. COL11A1 acts a tumor promotor of LUAD. A COL11A1 was positively associated with SPP1 in LUAD; B COL11A1 expression was significantly higher in LUAD tissues than normal tissues (*p <0.05, **p <0.01); C COL11A1 was significantly correlated to the TNM stage of LUAD; D patients with high COL11A1 expression had a poor OS than low COL11A1 patients. [file 12935_2022_2749_MOESM2_ESM.docx]
